# Surgical Repair of Aortic Atresia in an Adult

**DOI:** 10.1016/j.atssr.2025.08.007

**Published:** 2025-08-30

**Authors:** Misato Tokioka, Osamu Ishida, Koji Tsutsumi

**Affiliations:** 1 Department of Cardiovascular Surgery, National Defense Medical College, Tokorozawa, Japan

## Abstract

Aortic atresia is a rare congenital vascular anomaly that occurs in adults. Differentiating it from interrupted aortic arch without open surgery is difficult. In some patients with type A interrupted aortic arches, aortic atresia may actually be caused by aortic coarctation. A 38-year-old man was diagnosed with aortic atresia, aortic regurgitation with a bicuspid valve, and aortic root enlargement. The surgery was performed in 2 stages. First stage: Descending aortic replacement with partial cardiopulmonary bypass without cardiac arrest. Second stage: Bentall procedure with mechanical valve replacement. We discuss the pathophysiology of interrupted aortic arch and aortic atresia in adults.

Aortic atresia is characterized by the absence of luminal continuity between the aortic arch and descending thoracic aorta through an atretic segment, which appears like a fibrous cord.^1^^,^^2^ Aortic atresia in adults is a rare congenital obstructive malformation that is hypothesized to be an advanced form of severe aortic coarctation (CoA).^3^ Interrupted aortic arch (IAA) is characterized by the absence of continuity between the ascending and descending aorta. Type A IAA is the second most common type of IAA, accounting for 30%-40% of cases and is the most common type in adults.^4^ There is currently no reliable diagnostic method to accurately differentiate between IAA and aortic atresia except for open surgery.^1^ Some cases of IAA in adults may actually be aortic atresia.^3^ We present a case that was initially diagnosed as IAA but was later revealed to be aortic atresia based on surgical findings.

A 38-year-old man with refractory hypertension since childhood presented to our cardiology department with severe aortic regurgitation (AR). Computed tomography revealed a loss of continuity of the aorta distal to the left subclavian artery bifurcation and ductus arteriosus (Figure 1A). The brachiocephalic artery and left subclavian artery were markedly developed, with the bilateral internal thoracic and intercostal arteries and other collateral blood vessels extending to the lower extremities via the inferior abdominal wall (Figure 1B). Physical examination revealed high blood pressure (172/78 mm Hg) in both upper limbs and a weak pulse in both femoral arteries. Blood pressure pulse wave examination revealed a decrease on both sides (0.66/0.69). Echocardiography revealed a left ventricular ejection fraction of 58%, aortic root enlargement (Valsalva sinus, 49 mm), and severe AR with a bicuspid aortic valve (BAV) (Figures 1C, 1D). Our preoperative diagnosis was severe AR with a BAV, aortic root enlargement, and type A IAA. A 2-stage plan was then developed.

**Figure 1:**
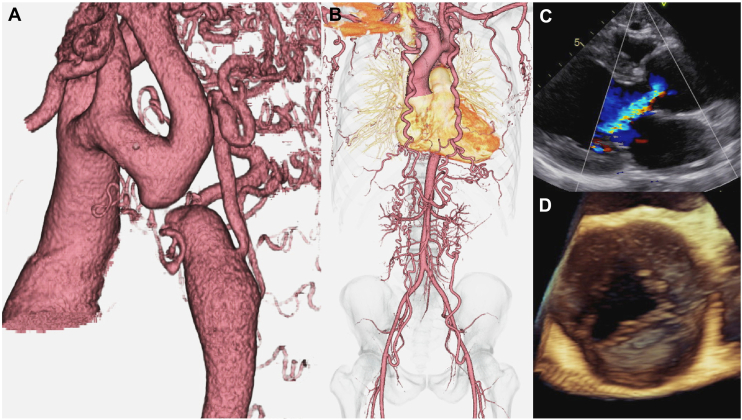
( A) Three-dimensional computed tomography shows loss of continuity of the aorta distal to the left subclavian artery bifurcation and ductus arteriosus and notably developed collateral circulation. (B) The brachial and left subclavian arteries are notably developed, with other collateral blood vessels to the lower limbs via the inferior abdominal wall. (C) Preoperative transthoracic echocardiography shows aortic root enlargement and severe aortic regurgitation. (D) Bicuspid aortic valve with raphe at the right and left coronary cusp identified by transesophageal echocardiography.

The initial surgery involved descending aorta replacement via a left fourth intercostal thoracotomy. During thoracotomy, bleeding from the intercostal arteries was severe and required careful monitoring. Gross findings revealed severe stenosis and fibrous tissue between the proximal and distal aortas, confirming the diagnosis of aortic atresia (Figure 2A). After clamping and incising the aorta, internal lumen continuity was completely lost distal to the ductus arteriosus (Figures 2B, 2C). When the aortic lumen was observed from the proximal side, independent cavities extending to the ductus arteriosus and distal aorta were found (Figure 2B). When observing from the distal aortic side, a cavity extending toward the proximal aorta was noted (Figure 2C). The wall of the aorta was thin, and no aneurysms were observed. We performed end-to-end anastomosis using an artificial graft (Figure 2D). Three months later, a second surgery was performed. The Bentall procedure with a mechanical valve was performed via median sternotomy. Postoperative computed tomography showed a reduction in the collateral blood vessels, such as the bilateral internal thoracic arteries. Blood pressure pulse wave examination revealed bilateral improvements (1.19/1.26).

**Figure 2:**
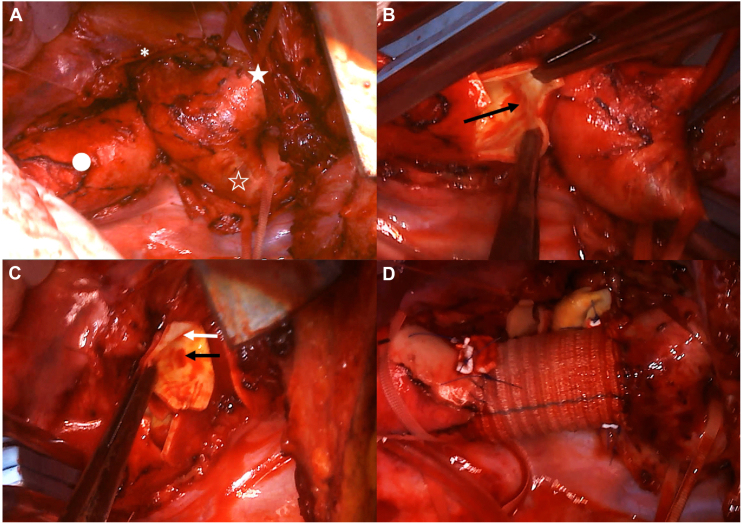
(A) After thoracotomy, the external continuity between the proximal aorta and distal aorta show severe stenosis and continuity with fibrous tissue. (★, proximal aorta; ☆, left subclavian artery; ●, distal aorta; ∗, ductus arteriosus.) (B) Observation from the distal side of the interruption reveals a cavity toward the proximal aorta (black arrow). (C) Observations from the proximal side of the interruption reveal two separate cavities in the distal aorta (black arrow) and ductus arteriosus (white arrow). (D) After descending aorta replacement with an artificial graft.

## Comment

**Figure 3:**
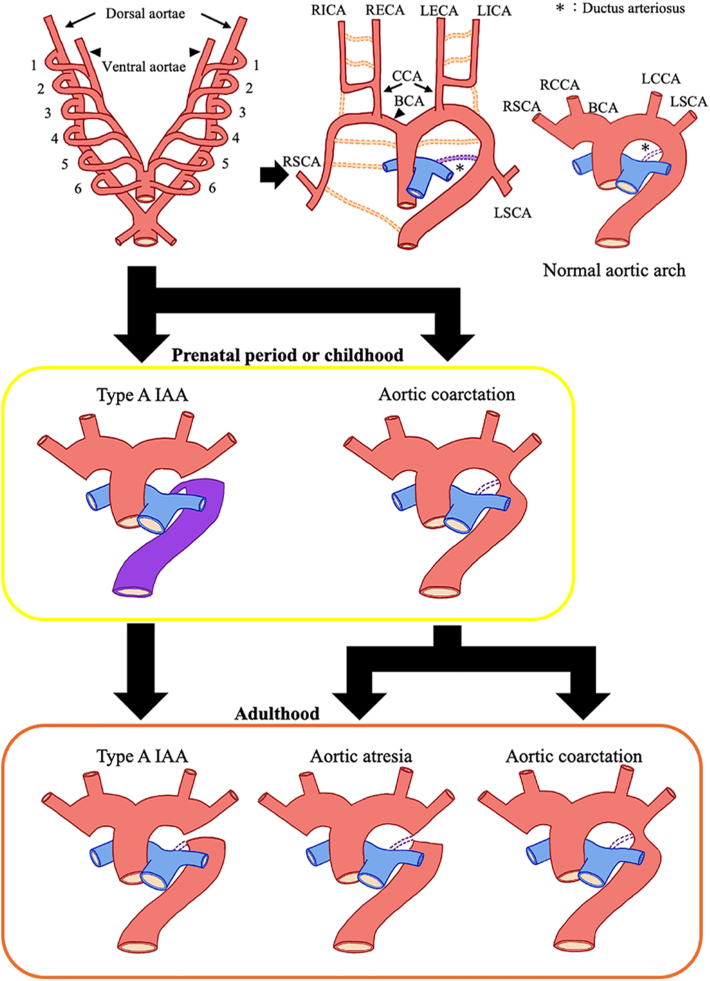
Original image diagrams of interrupted aortic arch, aortic coarctation, and aortic atresia in adults. (BCA, brachiocephalic artery; CCA, common carotid artery; IAA, interrupted aortic arch; LCCA, left common carotid artery; LECA, left external carotid artery; LICA, left internal carotid artery; LSCA, left subclavian artery; RCCA, right common carotid artery; RECA, right external carotid artery; RICA, right internal carotid artery; RSCA, right subclavian artery.)

IAA is rarely diagnosed in adults because it is usually diagnosed using fetal ultrasonography or after birth in the setting of circulatory failure or shock. To survive after birth, blood flow to the lower limbs must be maintained by the ductus arteriosus, and collateral circulation must be significantly developed to survive in adulthood.^5^ Aortic atresia in adults is considered a form of completely obstructed CoA due to aging.^1^ Approximately 44%-84% of cases of CoA are associated with congenital heart disease or aortic arch abnormalities.^4^ The complications of aortic atresia are similar to those of CoA and sometimes include AR with a BAV.^6^ Based on these findings, we propose a classification of IAA, CoA, and aortic atresia (Figure 3).

In this patient, preoperative assessment revealed type A IAA, but the continuity of the external lumen was uncertain. Intraoperative observations revealed that the aorta was interrupted at a point distal to the ductus arteriosus with fibrous continuity. To survive, patients with IAA require a patent ductus arteriosus to maintain blood flow to the lower limbs; however, this condition usually leads to fatal results after birth. Before surgery, blood flow to the lower body is maintained by abundant collateral circulation. However, collateral circulation is unlikely to be present at birth. Consequently, we diagnosed the patient with aortic atresia owing to the presence of BAV, aortic root enlargement, and fibrous continuity. We believe that this patient had CoA at birth that gradually progressed to aortic atresia over time; however, it is challenging to diagnose prior to surgery.

Type A IAA and aortic atresia have similar clinical symptoms and hemodynamics and are treated with revascularization.^7^ Revascularization methods include open surgery and percutaneous stent placement. Open surgery carries the risk of bleeding from the collateral vessels, but the outcome is usually stable after surgical repair. Percutaneous stent placement involves creating a hole in the IAA septum and inserting a covered stent; however, there have been reports of endovascular treatment-linked deaths from hemorrhagic complications.^1^ We performed descending aortic replacement and the Bentall procedure with a mechanical valve for aortic atresia and AR with a bicuspid valve and aortic root enlargement. The patient showed positive progression after the 2-stage surgical procedure. We consider open surgery as a more robust treatment.

In conclusion, we performed surgical treatment for aortic atresia diagnosed in an adult. Differentiating IAA from aortic atresia was challenging; however, surgical revascularization resulted in beneficial outcomes.
